# Rethinking tracheostomy care: cuffless tubes for patients with aspiration

**DOI:** 10.3389/fresc.2026.1737737

**Published:** 2026-06-11

**Authors:** Bettina Arca-Tschudi, Ludwig D. Schelosky, Paul Diesener, Svetlana Politz Geleva, Karsten Krakow

**Affiliations:** 1 VITREA Rehabilitation Clinic, Zihlschlacht, Switzerland; 2 Private University in the Principality of Liechtenstein (UFL), Triesen, Liechtenstein; 3 Klinische Neurologie, District Hospital Rohrbach, Rohrbach, Austria; 4 Department for Neurological Rehabilitation, Hegau-Jugendwerk, Gailingen, Germany; 5 Klinik für Neurologie, Cantonal Hospital of Münsterlingen, Münsterlingen, Switzerland

**Keywords:** airway protection, aspiration pneumonia, cuffless cannulas, dysphagia, neurological rehabilitation, non-ventilated, tracheostomized, tracheostomy tube management

## Abstract

**Introduction:**

Guidelines do not recommend cuffless tracheostomy tubes in patients at risk of aspiration, although there is no evidence supporting the protective effect of cuffed tubes against aspiration or pneumonia. The Zihlschlacht Neurological Rehabilitation Clinic has routinely implemented early transition to cuffless tubes to support rehabilitation, facilitate communication and improve quality of life. This study evaluates the impact of this approach on tracheostomy management and pneumonia rates.

**Methods:**

Retrospective analysis included 124 non-ventilated, tracheostomized neurological patients admitted between January 2019 and December 2022. Outcomes were pneumonia, aspiration, secretion burden, and tolerance of speaking valves or decannulation caps.

**Results:**

Of the 124 patients, 119 were admitted with cuffed tubes. Pneumonia was documented in 112 patients and aspiration in 77. All patients were switched to cuffless tubes within seven days, including those with severe aspiration. After conversion to cuffless tubes, suspected aspiration decreased from 29 to 13 and confirmed aspiration from 48 to 42 cases, totaling 55, while pneumonia fell to 14. Most patients tolerated speaking valves or decannulation caps immediately, required less suctioning, and showed a marked reduction in secretions.

**Discussion:**

Early conversion to cuffless tubes did not increase aspiration pneumonia and was associated with improved secretion management and easier airway care. These findings challenge the presumed protective role of cuffed tubes and support the feasibility of early cuffless use in neurological rehabilitation. Aspiration risk alone should not preclude early cuffless management. Prospective studies are needed.

## 1 Introduction

Patients with neurological impairment and severe deficits in airway protection often require intensive care and mechanical ventilation in the acute phase. For instance, 30%–80% of stroke patients experience dysphagia (1, 2). Following tracheostomy, dysphagia is diagnosed in 11% to 93% of cases (3, 4). However, preserved swallowing function is crucial for successful decannulation (5).

Decannulation following tracheostomy is a key part of the rehabilitation process. Despite its high clinical relevance, there are currently only a few evidence-based strategies for removing tracheostomy tubes in adult patients (6). There is an urgent need for reproducible, standardised protocols to guide clinical practice (7).

Although existing recommendations often advise against using cuffless tracheostomy tubes in patients at increased risk of aspiration (6), there is currently no reliable evidence that cuffed tubes prevent material from entering the lower respiratory tract or reduce the incidence of pneumonia (8). Microaspiration regularly occurs despite an intact cuff (9, 10). Studies show that fluid penetration into the subglottic space can lead to deeper aspiration along the cuff folds. The air insufflation of the cuff varies depending on the type of tube, and there is currently no uniform standard for optimal cuff pressure (11). Many models exceed the limit for potential tracheal damage at pressure values of around 25 cm H₂O, even when correctly positioned, yet this is common clinical practice (8).

Deflating the cuff and using speaking valves has a positive effect on swallowing physiology, as it restores subglottic pressure and promotes the coordination of coughing and swallowing (10). This improves airway protection, reduces the risk of aspiration and improves quality of life (10, 12). Several studies have demonstrated better functional outcomes in patients who are decannulated at an early stage (13).

To date, there are few guidelines on practical procedures for everyday clinical practice (6, 14, 15). At the Zihlschlacht Neurological Rehabilitation Clinic, a cuffless tracheal cannula is inserted upon admission for patients who are spontaneously breathing. This creates a decisive prerequisite for efficient decannulation in the further course of treatment. Tracheoscopy is essential for the optimal care of tracheostomy tubes and for the early transition from cuffed to cuffless tubes (16). Fibreoptic endoscopic evaluation of swallowing (FEES) is another important decision-making aid for ensuring a safe, complication-free decannulation process (6, 17). The present study aims to examine whether this approach offers advantages over the longer-term use of cuffed tracheostomy tubes.

## 2 Methods

**Figure 1:**
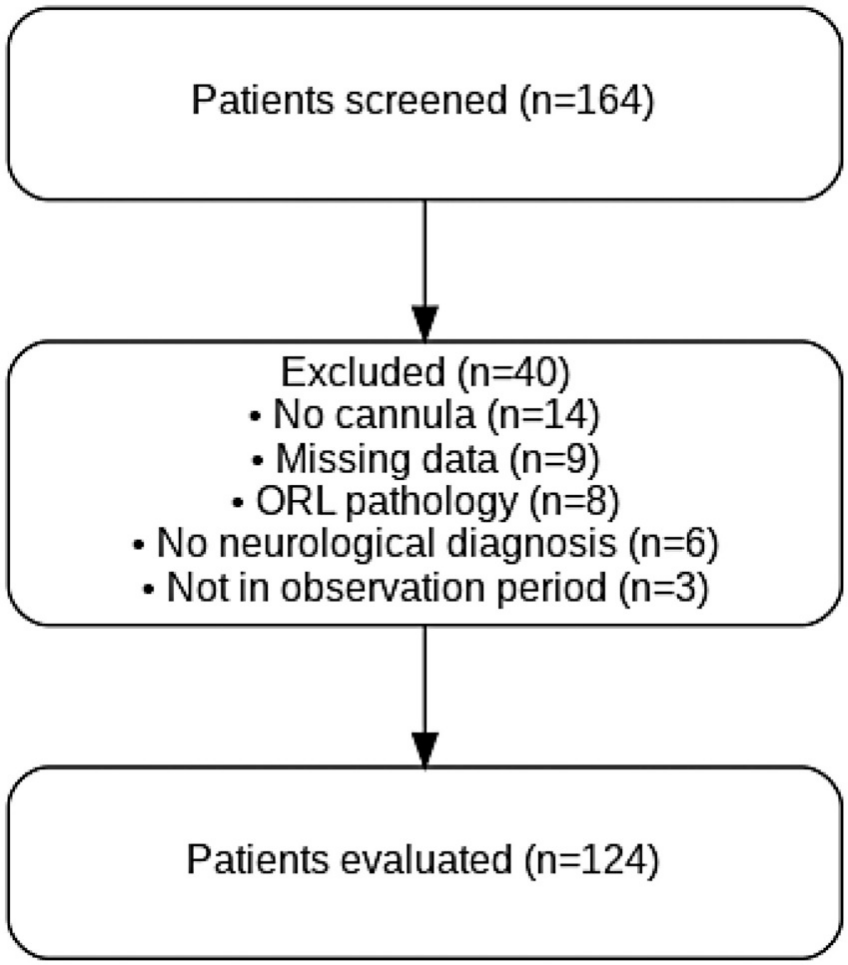
Inclusion and exclusion criteria.

At the Zihlschlacht Neurological Rehabilitation Clinic, data were collected retrospectively on all 164 non-ventilated patients admitted between January 1st, 2019 and December 31, 2022. Patients with an underlying neurological diagnosis, dysphagia and a tracheostomy tube on admission were included in the study. Patients without or with already removed tracheostomy tubes, those with poor prior clinical documentation, and individuals with non-neurological disorders were excluded (see Figure 1).

Upon admission to the rehabilitation clinic, all included patients underwent a clinical examination and a tracheoscopy. If necessary for cannula management, a FEES was performed in addition to the tracheoscopy. As part of the clinic's standard protocol, the cuff of the tracheostomy tube was deflated. Tolerance of a speaking valve and/or decannulation cap was tested based on clinical impression (i.e., resistance-free exhalation and inhalation) and switching to a cuffless tracheostomy tube was performed within seven days after admission even in cases where there was a risk of aspiration. During each tracheostomy tube change, all patients were continuously monitored, and the newly placed tube was subsequently inspected endoscopically. The switch to cuffless tracheostomy tubes was instituted because no evidence supported the continued use of cuffed tracheostomy tubes in spontaneously breathing patients. Moreover, this approach has long been the preferred practice at Zihlschlacht Neurological Rehabilitation Clinic and recent findings further underscore the lack of evidence for cuffed tracheostomy tubes (18). Furthermore, a clearly defined decannulation protocol is still lacking because there is no clinical-trial data or other robust evidence on which such guidance could be based (19).

All data were anonymised before analysis. Statistical analysis was employed to investigate the relationship between the early use of cuffless tracheostomy tubes and the incidence of pneumonia and other clinical outcomes. Pneumonia was defined based on clinical findings, including fever, characteristic radiographic changes, leukocyte counts >10,000 or <4,000/μL, elevated C-reactive protein levels, and the presence of purulent secretions (20).

The data were recorded using Excel 2024 and analysed using PSPP (GNU PSPP 2.0.0-g5b54d1). Descriptive statistics were calculated for all variables in the entire population (*n* = 124) and then again for patients (*n* = 77) who had suspected or endoscopically confirmed aspiration of saliva when fitted with cuffed tracheostomy tubes and were later fitted with cuffless tracheostomy tubes. For the comparisons, only post-admission pneumonia events were considered, and the same group of patients was analysed first with a cuffed tube and then with a cuffless tube using the Wilcoxon signed-rank test. The significance level was set at 5%.

## 3 Results

Fourteen patients without a tracheostomy tube were excluded. In nine patients, the hospitals where they had been treated previously provided insufficient data. Patients with structural respiratory disorders were also excluded (seven due to ORL tumours and one due to long-term tracheotomy). Six patients had no neurological admission diagnosis, and three patients were not included in the study period. This resulted in a patient group of 124 individuals with underlying neurological diseases, tracheostomy tubes, and neurogenic dysphagia (see Table 1). The patient characteristics and details of tracheostomy tube care are shown in Table 2.

**Table 1:** Primary diagnosis.

Primary diagnosis	Frequency	Percentage
Critical illness polyneuropathy (CIP)	24	19.4%
Cerebral ischemia	18	14.5%
Intracerebral hemorrhage (ICH)	21	16.9%
Subarachnoid hemorrhage (SAH)	6	4.8%
Trauma (traumatic brain injury/TBI)	12	9.7%
Status epilepticus	3	2.4%
Multiple sclerosis (MS)	4	3.2%
Inflammatory (GBS, meningitis, autoimmune)	9	7.3%
Hypoxic encephalopathy	18	14.5%
CNS tumor	4	3.2%
Other	5	4.0%
Total	124	100.0%

**Table 2:** Patient characteristics.

Characteristic	Value		
Age at admission (a) (mean, range, SD)	64.08 (20–85, SD 13.15)		
Sex (m/f)	80 (64.5%)/44 (35.5%)		
Length of stay (d) (mean, range, SD)	110.45 (21–373, SD 60.7)		
Reason for tracheostomy tube	Ventilation	82	66.1%
	Dysphagia	31	25.0%
	Miscellaneous	11	8.9%
Tracheostomy tube on admission	Cuffless	5	4.0%
	Cuffed	119	96.0%
Feeding tube on admission	None	1	0.8%
	NG-tube	26	21.0%
	PEG/PEJ tube	97	78.2%
Tracheostomy tube at discharge	None	95	76.6%
	Yes	29	23.4%
Reason for tracheostomy tube upon discharge	Tracheostomy suction port	24	19.4%
	Airway secured via tracheostomy tube	5	4.0%
Feeding tube upon discharge	None	67	54.0%
	NG-tube	0	0%
	PEG/PEJ tube	57	46.0%
Duration with cuffed tracheostomy tube (d) (mean, range, SD)	40.2 (13–144, SD 26.68)		
Change to cuffless tube after duration (d) (mean, range, SD)	13.74 (1–111, SD 16.9)		
Duration with cuffless tracheostomy tube (d) (mean, range, SD, Median IQR)	38.18 (1–195, SD 35.28, Q1 16, Median 27.5, Q3 47.5, IQR 31.5)		
Total duration with tracheostomy tube (d) (mean, range, SD)	68.93 (1–338, SD 48.01)		

Of the 124 patients included in the data collection, 119 had a cuffed tracheostomy tube on admission. In 112 cases, pneumonia had been documented prior to admission (112) and during the rehabilitation stay (11). The presence of clinical suspicion or endoscopic confirmation of aspiration was documented in 29 and 48 cases, respectively, amounting to a total of 77 cases. In accordance with the established clinic protocol, all 119 patients were transitioned to cuffless tubes of reduced diameter. Following the aforementioned change, aspiration was suspected or confirmed in 13 and 42 cases, respectively, for a total of 55 cases, with (aspiration) pneumonia occurring in a total of 14 cases.

The majority of patients showed a satisfactory tolerance to the cap or speaking valves, with no significant increase in the necessity for suctioning. In 76.6% of cases, decannulation was successfully performed after a period of four days without the need for suctioning. In 29 patients (23.4%) decannulation was not performed. This subgroup included 24 patients (19.4%), who required ongoing suctioning due to inadequate cough effectiveness, and 5 patients (4.0%) required a secure airway because of airway stenosis.

Following a 40.2-day period (13–144, SD 26.68), and 13.74 days subsequent to admission to rehabilitation (1–111, SD 16.9), the tracheostomy tubes were replaced with a smaller, cuffless tracheostomy tube model, in accordance with the clinic protocol. The cuffless tubes remained in place for a mean duration of 38.18 days (1–195, SD 35.28, Q1 16, Median 27.5, Q3 47.5, IQR 31.5). Following the transition to cuffless tubes, the incidence of pneumonias (only pneumonias occurring after admission to rehabilitation were included in this analysis) did not exhibit a higher frequency in comparison with that observed with cuffed tubes [Z = −0.47, Asymptotic Sig. (2-sided) = 0.635] Furthermore, saliva aspiration exhibited a substantial decrease [Z = −4.12, Asymp. Sig. (2-sided) < 0.001]. The suction frequency and the volume of secretions, with frequency based on the number of suctioning episodes per day and secretion volume derived from the nursing staff's documented keywords, were significantly reduced. The suction frequency decreased significantly [Z = –8.95, Asymp. Sig. (2-sided) < 0.001], consistent with the significant reduction in secretion volume in patients with cuffless tubes [Z = –9.23, Asymp. Sig. (2-sided) < 0.001]. Despite the limited objectivity of the data, these measures represented the best available indicators.

**Figure 2:**
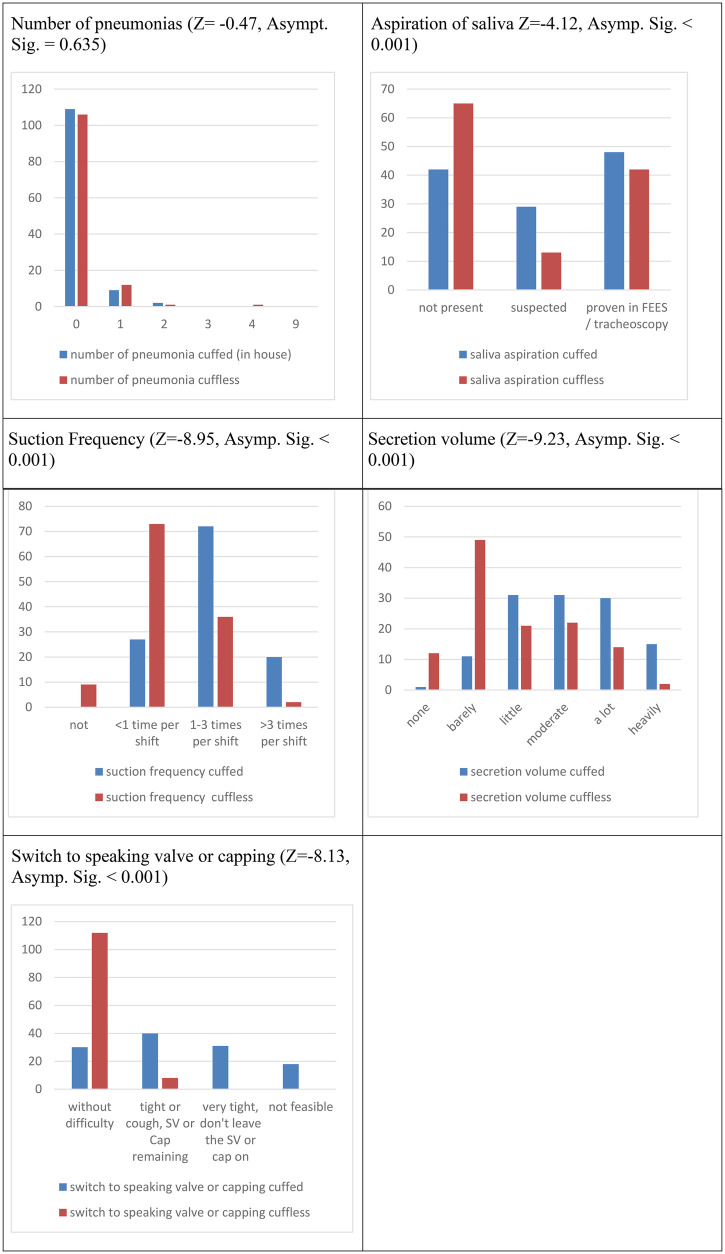
Comparison of cuffed and cuffless tubes in the overall group.

Treatment with a speaking valve or capping was significantly easier in patients with cuffless tubes than in patients with cuffed tubes [Z = −8.13, Asymp. Sig. (2-sided) < 0.001] (see Figure 2).

A subgroup of 77 patients in which the subjects were known to aspirate their saliva while using cuffed tracheostomy tubes was evaluated separately once again. Following the change, the incidence of pneumonia remained unchanged in this subgroup when compared against treatment with cuffed tubes [Z = −0.47, Asymp. Sig. (2-sided) = 0.635]. Like the overall group, patients in this subgroup also required less frequent suctioning after the change than before [Z = −7.35, Asymp. Sig. (2-sided) < 0.001]. These 77 patients, who had previously aspirated more frequently, produced less secretion after switching to a cuffless tracheostomy tube [Z = –7.38, Asymp. Sig. (2-sided) < 0.001] and tolerated the use of a speaking valve or capping significantly better, similar to the overall group after switching to the cuffless tracheostomy tube [Z = –6.57, Asymp. Sig. (2-sided) < 0.001].

To evaluate whether differences in the duration of cuffed (mean 40.2 days) and cuffless (mean 38.18 days) tracheostomy tube use might influence pneumonia incidence, Pearson correlation coefficients were calculated. No association was observed between the total number of pneumonias and the overall duration of tracheostomy cannulation (*r* = 0.162, *p* = 0.0839) or between pneumonias and the duration of cuffless cannulation (*r* = 0.121, *p* = 0.259). In contrast, a positive correlation was found between the number of pneumonias occurring with cuffed tubes and the duration of cuffed cannulation (*r* = 0.364, *p* < 0.0001), suggesting that longer periods with a cuffed tube may be associated with higher pneumonia rates, although this relationship may also reflect differences in the patient's underlying disease severity.

## 4 Discussion

This retrospective analysis of 124 patients with underlying neurological diseases, tracheostomy tubes and neurogenic dysphagia shows that switching from cuffed to cuffless tracheostomy tubes does not increase the risk of aspiration pneumonia. This contradicts the common assumption that the cuff reliably protects against aspiration and pulmonary infection, thus confirming earlier studies (9, 10, 21). The study was conducted monocentrically in a neurological rehabilitation clinic. The generalizability of the results to acute hospitals or other clinical facilities is not intended. Conversely, cuffless tracheostomy tubes offer several advantages. The volume of secretions, and consequently the frequency of suctioning, decreased significantly in the present study. Importantly, the absolute amount of aspirated saliva cannot be reliably quantified once patients are able to cough effectively; material that would otherwise accumulate above the cuff is now removed spontaneously. Restoring natural airflow through the airways normalizes respiratory air climatization and restores the natural self-cleaning of the bronchial tree. Airflow through the mouth and nose stimulates sensory receptors, promotes secretion mobilisation and improves breathing-swallowing coordination (22). This creates a functional-physiological balance that enables patients to cough effectively and eliminate aspirated saliva by themselves. Therefore, the use of cuffless tubes appears not only to be safe, but even beneficial for physiological airway care, which is consistent with the observations of Pryor et al. (22).

Another key finding is that speaking valves and caps are significantly easier to use on cuffless tracheostomy tubes. This improvement has communicative and psychosocial advantages as well as functional consequences for rehabilitation. Using a speaking valve enables phonation and increases sensory stimulation in the laryngeal and oropharyngeal areas. It also promotes the restoration of the cough and swallowing reflexes and improves quality of life (12, 22, 23). Effective coughing is only possible with a cuffless tracheostomy tube combined with a speaking valve or cap. In this case, expiratory airflow is channelled into the upper airways. This may lead to more efficient secretion mobilization and could reduce the risk of respiratory complications. Cough efficiency and sensory reactivity in cases of dysphagia are considered decisive parameters for successful cuff deflation and decannulation (22, 23). These mechanisms may help explain the clinical improvements observed in a subgroup of 77 patients who initially aspirated large amounts of saliva when the cannula was cuffed. Following the switch to cuffless tracheostomy tubes, there was no increase in pneumonia within this subgroup. Additionally, there was a significant reduction in the volume of secretions and the frequency of suctioning, as well as an improvement in tolerance of the speaking valve or cap.

The perceived sense of security associated with the use of cuffed tracheal cannulas in everyday clinical practice is therefore questionable. In fact, blocking the airflow through the upper airways can lead to desensitisation of the oropharyngeal mucosa, weakening reflexive protective mechanisms such as coughing and swallowing in the long term (23). This is further supported by the finding that longer durations with a cuffed tracheostomy tube were associated with a higher number of pneumonia episodes. In light of this, it only makes sense to maintain a cuffed tracheal cannula in non-ventilated neurologically ill patients if there are compelling respiratory reasons to do so, such as maintaining ventilation pressure. For all other patient groups, the results suggest cuff deflation early and using cuffless systems. These enable more physiological breathing and communication, and reduce the risk of tracheal complications such as ulcers, tracheomalacia or stenosis, which are associated with high cuff pressures (6). These findings underscore the need for prospective studies to develop evidence-based algorithms for tracheostomy management in neurological patients, including stratification by neurological diagnosis and cough effectiveness, regarding the use of cuffed vs. cuffless tubes in spontaneously breathing patients.

## 5 Conclusion

In this monocentric retrospective cohort of 124 neurologically impaired, non-ventilated tracheostomized patients, early cuff deflation and transition to cuffless tracheostomy tubes were not associated with an increased risk of aspiration pneumonia. Our findings suggest that aspiration should not preclude cuffless tubes in non-ventilated rehabilitation patients. Instead, cuffless tubes were linked to reduced secretion burden, fewer suctioning requirements, and improved tolerance of speaking valves or capping, thereby facilitating more physiological airflow, speech, enhanced sensory stimulation, and more effective cough and swallowing function. These findings challenge the assumption that cuff inflation reliably prevents aspiration and highlight the potential functional and rehabilitative advantages of cuffless systems in spontaneously breathing neurological patients. Nevertheless, the study's retrospective design, lack of a control group, and single-center setting limit generalizability, particularly to acute care environments. Prospective multicenter studies are needed to validate these results and to refine evidence-based algorithms for tracheostomy management in this vulnerable patient population, with data collection planned around clearly defined variables to minimize confounding and allow robust statistical analysis.

## Data Availability

The datasets presented in this article are not publicly available but can be obtained from the corresponding author upon reasonable request (Bettina Arca-Tschudi; bettina.arca-tschudi@outlook.com).
