# Supplementary figures and images for: Rethinking tracheostomy care: cuffless tubes for patients with aspiration
Source: Front Rehabil Sci. 2026 Jun 11;7:1737737. doi: 10.3389/fresc.2026.1737737 (PMC13294461; doi:10.3389/fresc.2026.1737737)

Supplementary Figure 1. Odds Ratio

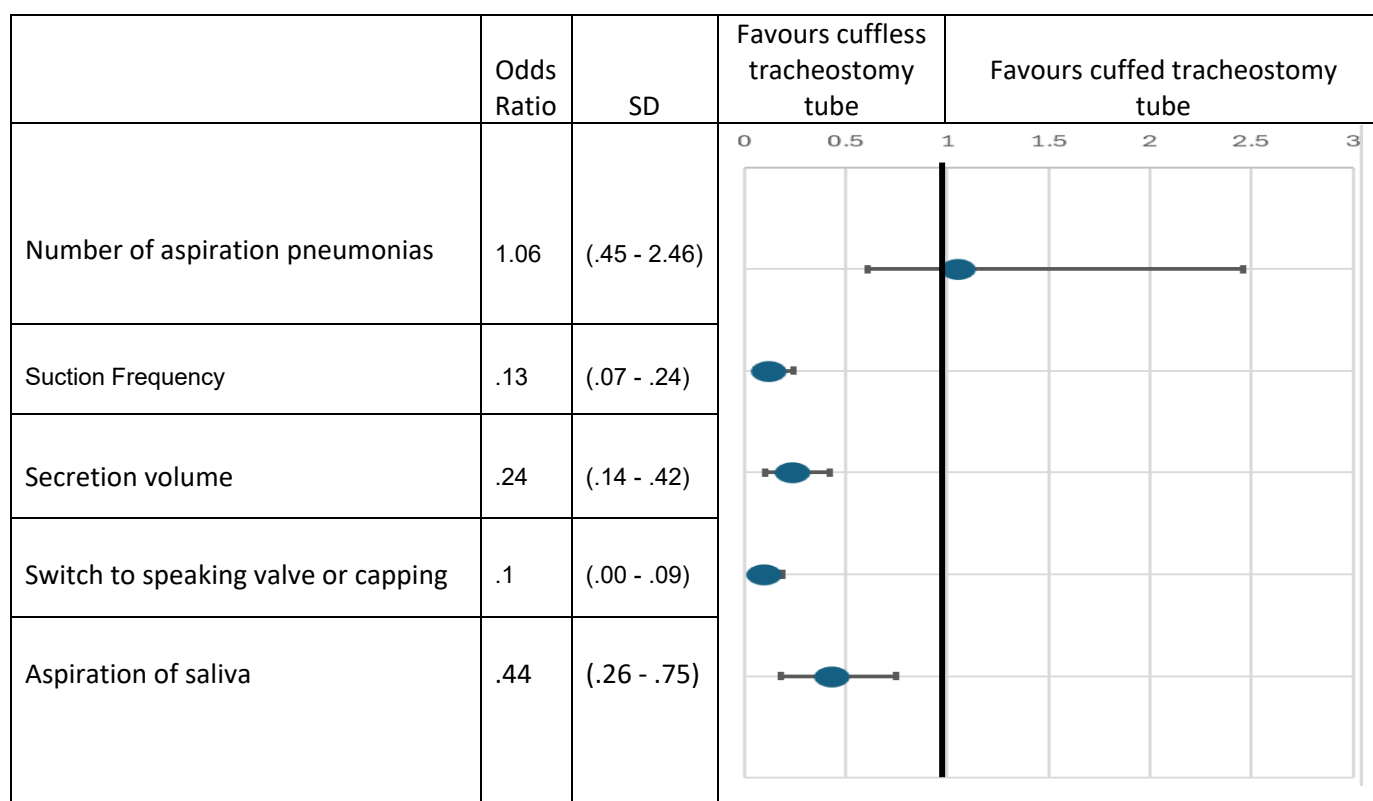

Supplement: Supplementary file 1 [file image1.pdf]
